# Screening for transcriptomic associations with Swine Inflammation and Necrosis Syndrome

**DOI:** 10.1186/s12917-024-04469-y

**Published:** 2025-01-17

**Authors:** Katharina Gerhards, Sabrina Becker, Josef Kuehling, Mirjam Lechner, Hermann Willems, Robert Ringseis, Gerald Reiner

**Affiliations:** 1 Department of Veterinary Clinical Sciences, Clinic for Swine, Justus-Liebig-University, Frankfurter Strasse 112, D-35392 Giessen, Germany; 2 UEG Hohenlohe, Am Wasen 20, 91567 Herrieden, Germany; 3 Institute of Animal Nutrition and Nutrition Physiology, Justus Liebig University Giessen, Heinrich-Buff-Ring 26-32, 35392 Giessen, Germany

**Keywords:** Inflammation, Necrosis, Swine, Animal welfare, Metabolism

## Abstract

**Background:**

The recently identified swine inflammation and necrosis syndrome (SINS) affects tail, ears, teats, coronary bands, claws and heels of affected individuals. The primarily endogenous syndrome is based on vasculitis, thrombosis, and intimal proliferation, involving defence cells, interleukins, chemokines, and acute phase proteins and accompanied by alterations in clinical chemistry, metabolome, and liver transcriptome. The complexity of metabolic alterations and the influence of the boar led to hypothesize a polygenic architecture of SINS. This should be investigated by a transcriptome study. For this purpose, the three to five least affected (SINS-low) and most SINS affected (SINS-high) 3d-old piglets, each of three boars, a relatively SINS stable Duroc boar (DU), a relatively stable Pietrain boar (PI+) and a highly susceptible Pietrain boar (PI-) were selected from 27 litters of mixed semen to minimize environmental effects.

**Results:**

A genome-wide expression experiment revealed a huge set of differentially expressed genes that are involved in vasculitis, inflammation and necrosis, keratinization and erythrocyte epitopes. Among them were *CRP*, *GYPA*, *S100A12*, and *LIPK*. The results confirm and complement previous studies to this topic.

**Conclusions:**

The results confirm the outstanding importance of defence in the context of SINS. At the same time, for the first time, there is evidence for a direct involvement of the keratinisation capacity of the skin and various epitopes of the erythrocyte membrane, which seem to be associated with the severity of SINS. These genes could serve to clarify the pathogenesis of the syndrome and to develop diagnostic tools in future studies.

**Supplementary Information:**

The online version contains supplementary material available at 10.1186/s12917-024-04469-y.

## Background

Observations of clinical signs of inflammation at the base of the tail, tail tip, ears, teats, navel, coronary bands, heels and claw wall in piglets, weaners and fatteners led to the establishment of Swine Inflammation and Necrosis Syndrome (SINS) [1–6]. In this context, bristle loss, then swelling and redness, and later rhagades, exudation hemorrhage and necrosis were observed. Such changes lead to painful conditions and thus to concerns about animal welfare in pig production [7, 8]. The fact that the syndrome can occur simultaneously in so many different body parts [2, 4, 5], that suckling piglets can already show signs at the time of birth, when mechanical irritation and biting are still excluded [4], and the histopathological evidence of inflammatory changes starting from blood vessels, with intact epidermis [3, 4] argue for a primary endogenous genesis of the syndrome. There is also evidence on farm and from experiments of an interaction between SINS severity and environmental effect: the more severe the inflammatory symptoms, the more susceptible the affected animals are to mechanical stress, and the more severe the environmental stimuli (e.g., unfavourable floor), the more pronounced the expected SINS signs [1]. This explains why the signs of SINS can vary significantly depending on the existing environmental factors in different herds [9]. Nevertheless, none out of 646 piglets was completely free from signs, and 40% of piglets were affected in at least five of seven body parts examined [5].

The clinical signs of inflammation concerning the tail could be explained by vasculitis, intima proliferation, thrombosis, edema, hyperaemia and the accumulation of inflammatory cells perivascularly, despite intact epidermis [3, 4]. Bristle loss was also associated with inflammatory processes in the deeper parts of the hair follicles [3, 4]. Forty to 80% of neonatal piglets were affected by haemorrhages of the claw wall, coronal inflammation, redness of heels, bristle loss, and redness of the tail and ears. The inflammation could be characterized by granulocytes in considerable numbers, macrophages, and lymphocytes, indicating an onset of inflammation at least four days before birth [10], while the piglets were not older than two hours [4].

Clinical and histopathology are not the only findings. They are accompanied by massive alterations in clinical chemistry [11], of the metabolome and the liver transcriptome [9]. In these studies piglets had SINS scores between 0 and 2.25 in the SINS-low group (piglets with lowest SINS scores) and between 8.25 and 14.5 in the SINS-high group (piglets with highest SINS scores), but blood count and metabolic parameters were still largely unaffected in three-day old suckling piglets by SINS. Nevertheless, suckling piglets with SINS had significantly lower total protein and globulin levels and as a major finding, significantly increased C-reactive protein (*CRP*) levels in blood serum [11]. Serum levels elevated under SINS were associated with increased expression of *CRP* in the liver. At the same time, the expression of haptoglobin (*HP)*, intercellular adhesion molecule 1 (*ICAM1)*, interleukin 6 (*IL6)*, sodium oxyd dismutase 1 (*SOD1)*, fibroblast growth factor 21 (*FGF21)* and tumor necrosis factor (*TNF)*, were increased and interleukin 8 (*IL8)* was decreased in the liver. A series of pro-inflammatory genes and genes involved in stress response were up-regulated, among them were glycophorin A (*GYPA)* and Dimethylaniline monooxygenase 5 (*FMO5)*. C-C motif chemokine ligand 2 (*CCL2)* and other genes were significantly down-regulated [9].

Field experience and experimental studies demonstrate the influence of sow [3] and boar [5] genetics on piglet SINS grade under identical housing conditions. In the study by Kühling et al. [5], the sows were inseminated with mixed semen from the two boars whose effects on the offspring should be compared.

SINS scores in offspring from Duroc boars (DU) were significantly lower (4.87 ± 0.44) than those in offspring from Pietrain boars (DU) (10.13 ± 0.12). Based on their progeny, Pietrain with minimum and maximum sensitivity to SINS could be identified. The cumulative percentage of three-day-old piglets from Duroc boars affected by SINS signs at the tail base was 43.5%. The corresponding values for the progeny of the best Pietrain boars (PI+) was 92.1 and that of the poorest Pietrain boars (PI-) was 199.6. Exudation and necrosis occurred only in progeny of favourable (4.4%) and unfavourable Pietrain boars (20.1%), but not in progeny of Duroc boars. Total SINS scores in the offspring of the best Pietrain boars was almost 40% lower than that of offspring in the poorest Pietrain boars.

The present study was conducted to verify differentially expressed candidate genes under conditions of altered husbandry and genetics [9] using the progeny of three boars previously classified as extreme [5] and to generate further insights into the association of SINS with alterations in liver gene expression.

## Results

### Phenotypes

More than 86% of the total 236 three-day-old suckling piglets already showed swelling and bleeding of the heels and haemorrhages in the claw wall. The ears showed vein congestion in 86% and a shiny surface and bristle loss in over 65% of the animals. About 40–60% of the piglets showed swelling and redness of the tail base, venous congestion at the teats and signs of inflammation at the coronary bands of the front and hind limbs. Over 20% of the piglets had lost bristles at the tail base, scab formation at the tail tip and swelling at the teats. The more severe symptoms, such as haemorrhages and necroses, were only detectable in 4 to 8% of the piglets at this age.

Overall, average SINS scores for DU, PI + and PI- boar progeny were 10.4, 12.7 and 13.8, respectively. The confidence intervals of the boar progeny were clearly separated and ranged from 9.3 to 11.6, 12.1–13.2 and 13.2–14.4, respectively, for DU, PI + and PI-. The high variability of SINS signs made it possible to select piglets with extremely low and highly pronounced SINS signs from each of the three boar progenies for the transcriptome study.

Thus, at the level of the selected extreme animals, no more significant differences were detectable between the boars, while the piglets of each boar progeny group differed highly significantly between SINS-low and SINS-high in all body parts as well as in the SINS score (Table 1). There was no significant effect of the sex in this cohort of piglets. The variance explained by the boar was therefore only between 2 and 20%, while the SINS group explained between 28 and 91% of the phenotypic variance. The closest association to the boar was found for the ear score. Regarding SINS group, the highest correlation was found for tail base score, coronary band score, claw wall score, and total SINS score.

**Fig. 1:**
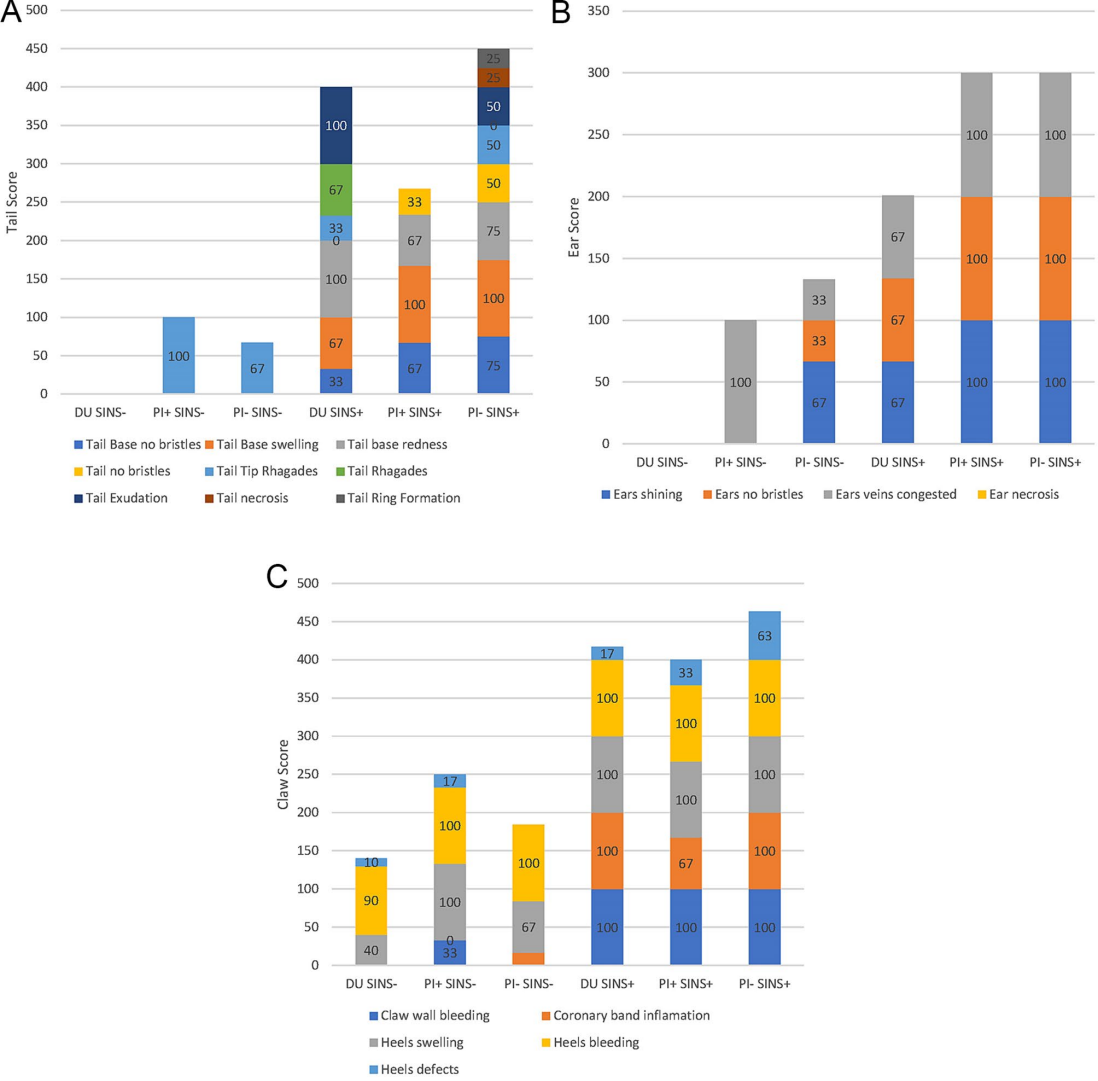
Stacked bar charts representing phenotypic scores with regard to boar (DU, PI+, PI-) and SINS status (low [-]: Z-scores between − 13.86 and − 5.39 vs. high [+]: Z-scores between 4.18 and 12.83). **A**) Tail score; **B**) Ears; **C**) Claws

The prevalence of the most important individual findings are shown in Fig. 1. In the SINS-low piglets, there were no problems at all on the tail of the Duroc offspring and also in the Pietrain offspring only bristle loss at the base of the tail was observed. In contrast, the scores in the SINS-high groups were high. The selected offspring of the PI + boar tended to perform best. They showed a maximum of bristle loss at the tail tip, while the DU offspring showed raghades and exudation, and the PI offspring even showed tail necrosis and ring formation (Fig. 1A).

A similar picture emerged for the ears of the piglets. Here, too, the changes increased considerably under SINS. However, the DU offspring were always less affected than the PI offspring (Fig. 1B). Ear necrosis was not observed in any case.

The claws including coronary bands and heels were also significantly more affected under SINS than in the SINS-low group. This was especially due to the occurrence of claw wall bleeding and inflammation of the coronary bands (Fig. 1C).

In summary, the results of the progeny of all three boars thus showed considerable differences in expression with regard to the SINS signs. In the following, we investigated whether and which genes are differentially expressed in the liver with regard to the SINS groups and whether additional boar-specific expression differences can be detected.

**Table 1:** Scores (Mean ± SE after Z-transformation) of the different body parts and SINS score by SINS (low vs. high) and boar breed (DU, PI^+^, PI^−^)

	SINS	DU	PI^+^	PI^−^	*R* ^2^_Boar_	*R* ^2^_SINS_	*P*_Boar_	*P*_SINS_
Ear Score	low	-2.15ad* ± 0.39	-1.25b ± 0.51	-0.94 cd ± 0.51	0.198	0.566	(0.053)	< 0.001
	high	-0.34a ± 0.51	1.17d ± 0.51	1.24abc ± 0.44				
Face Score	low	-0.62a ± 0.52	-0.24 ± 0.67	-0.88b ± 0.67	0.099	0.312	n.s.	0.005
	high	1.03 ± 0.67	0.4 ± 0.67	1.19ab ± 0.58				
Tail Tip Score	low	-0.67ab ± 0.41	0.29c ± 0.53	-0.03d ± 0.53	0.062	0.281	n.s.	0.008
	high	1.24a ± 0.53	-0.03e ± 0.53	1.95bcde ± 0.46				
Tail Base Score	low	-0.99abc ± 0.28	-0.99def ± 0.36	-0.99ghi ± 0.36	0.056	0.762	n.s.	< 0.001
	high	0.76adg ± 0.36	1.05beh ± 0.36	1.19cfi ± 0.31				
Teat Score	low	-0.64ad ± 0.41	-0.73b ± 0.52	-0.58c ± 0.52	0.027	0.386	n.s.	0.002
	high	0.16 ± 0.52	1.49abc ± 0.52	0.71d ± 0.45				
Claw Wall Score	low	-2.76abc ± 0.15	-1.7abc ± 0.2	-2.76abc ± 0.2	0.037	0.909	n.s.	< 0.001
	high	0.44a ± 0.2	0.44b ± 0.2	0.44c ± 0.17				
Coronary Band Score	low	-1.24ab ± 0.25	-1.24c ± 0.32	-0.88d ± 0.32	0.047	0.734	n.s.	< 0.001
	high	1.25acd ± 0.32	0.18acd ± 0.32	0.89bcd ± 0.28				
Heels Score	low	-1.72ad ± 0.6	-0.92b ± 0.77	-1.44c ± 0.77	0.019	0.497	n.s.	< 0.001
	high	0.3d ± 0.77	1.16ac ± 0.77	1.77abc ± 0.67				
SINS Score	low	-10.78aef ± 1.04	-6.77ae ± 1.34	-8.51bcd ± 1.34	0.017	0.892	n.s.	< 0.001
	high	4.83be ± 1.34	5.85ac ± 1.34	9.39df ± 1.16				

DU: offspring from Duroc boar; PI^+^: offspring from SINS stable Pietrain boar; PI^−^; offspring from SINS-susceptible Pietrain boar; R^2^: Coefficient of determination; P: significance; n.s.: not significant. Z-transformed SINS scores in SINS-low and SINS-high groups varied from − 13.86 to -5.39 and from 4.18 to 12.83, respectively

*: For each body part, six values (three boars in low or high SINS status were compared. Values with the same letter were statistically significant (*P* < 0.05)

### Transcriptomics

83 genes were differentially expressed with at least 2-fold difference between piglets with high and low SINS grades (*P* ≤ 0.05) (Supplemental Table 1). The expression of 19 genes was down-regulated by SINS, that of 54 genes was up-regulated. 70 differential expressions between SINS grades were found in Pietrain, 26 in Duroc progeny and 3 in both, *GYPA*, N-acylsphingosine amidohydrolase 2 (*ASAH2)*, and solute carrier family 16 member 6 (*SLC16A6)*. Table 2 shows a section with 42 genes that show the strongest expression differences with regard to SINS-low and SINS-high. Thirtysix of them were statistically significant at *P* ≤ 0.05 in certain boar groups and 9 further genes tended to be significant (*P* ≤ 0.1). Among them are *GYPA*, *CRP*, transmembrane protein 52B (*TMEM52B)*, S100 calcium binding protein A12 (*S100A12)*, hemogen (*HEMGN)*, *CCL2* und vascular endothelial growth factor A (*VEGFA)*.

**Fig. 2:**
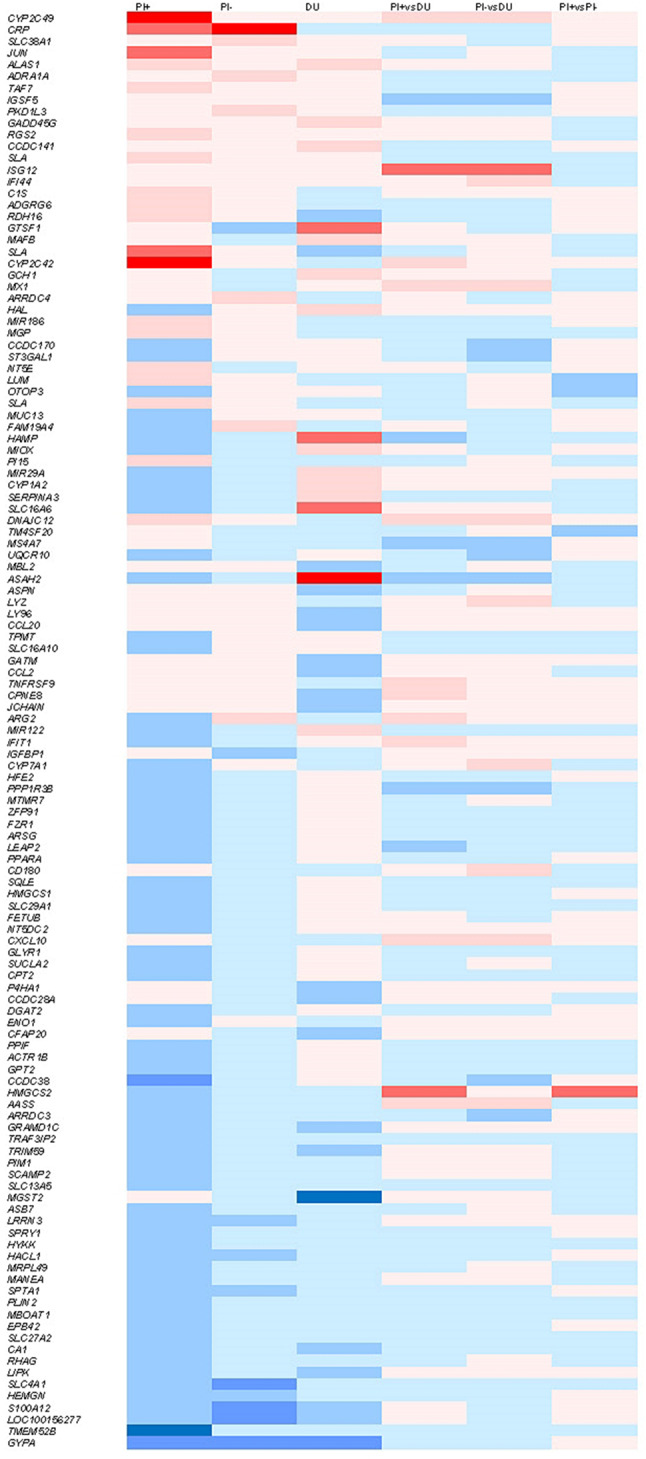
Heatmap of differentially expressed genes. Column 1: offspring from PI + boar (piglets with low SINS scores vs. piglets with high SINS scores). Column 2: the same for offspring from PI- boar. Column 3: the same for DU boar. Columns 4 to 6: comparison between any piglets (SINS-low [z-scores between − 13.86 and − 5.39] and SINS-high [z-scores between 4.18 and 12.83]) of the different boars. Fields are red, if the gene is upregulated in the second group (e.g. in SINS-high offspring from PI + boar in column 1); they are blue, if upregulated in the first group. Colour intensity increases in steps from 1-1.9-fold, 2-2.9-fold, 3-fold to > 4-fold, and − 1 to -1.9-fold, -2 to -2.9-fold, -3-fold, and < -4-fold, respectively

**Fig. 3:**
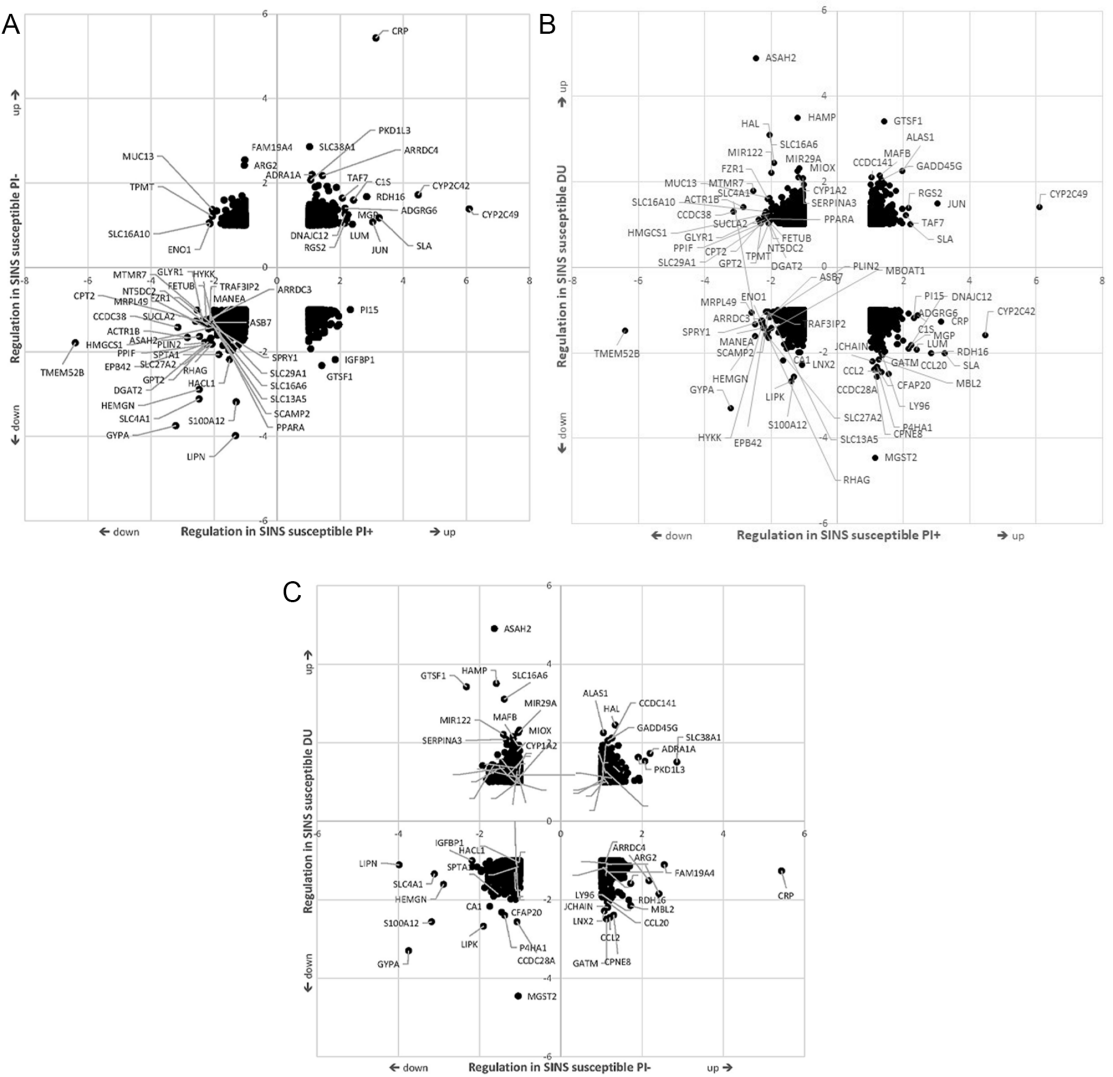
Double volcano plots comparing gene regulation in offspring from different boars. Units of X- and Y-axis are fold-change of the delogarithmised data. **A**) comparison between PI + and PI- offspring, both with high SINS scores [z-scores between 4.18 and 12.83]; **B**) comparison between PI + and DU offspring, both with high SINS scores; **C**) comparison between PI- and DU offspring, both with high SINS scores

Differences between piglets with low and high grades of SINS were most striking in PI + and DU boars, but less pronounced in PI- boar offspring (Fig. 2). There was a distinct difference between genes differentially expressed in SINS-low and SINS-high groups within boars (columns 1 to 3) and genes differentially expressed between boars, independent of the SINS score. Offspring of the three boars were compared in double vulcano plots, where any gene was located according to its fold-change in SINS-high piglets as compared to the SINS-low piglets of the two offspring groups (Fig. 3A: SINS susceptible PI + vs. SINS susceptible PI-; Fig. 3B: SINS susceptible DU vs. SINS susceptible PI+; Fig. 3C: SINS susceptible DU vs. Sins susceptible PI-). All genes with fold-change of > = 2 in at least one of the offspring group are named in the figures. The upper right quadrant shows genes up-regulated in the presence of SINS in both boars’ offspring group. *CRP* is the most prominently up-regulated gene in the offspring of both PI-boars, but not in DU. Swine lymphocyte antigen (*SLA*) and Jun proto-oncogene (*JUN*) are up-regulated in PI + offspring only. Cytochrom p450 c42 and c49 (*CYP2C42* and *CYP2C49)* are up-regulated in both PI boar offspring, but not in DU (< 2-fold). 5’-Aminolevulinate Synthase 1 (*ALAS1)*, solute carrier family 38 member 1 (*SLC38A1)* and others are upregulated in PI- and DU, but not in PI+. More than the up-regulated genes are down-regulated in SINS. The most prominent *G*enes in any offspring are *GYPA* together with *HEMGN* and solute carrier family 4 member 1 (*SLC4A1)*. Erythrocyte membrane protein band 4.2 (*EPB42)* and *TMEM52B* are further down-regulated in PI+. *S100A12* is strictly down-regulated in PI- and DU offspring. Few genes are up-regulated in PI + and at the same time down-regulated in PI- or vice-verse. However, many genes are regulated in different direction when comparing PI + and DU.

**Table 2:** The 44 genes with the highest fold-change in the liver transcriptome of the different boars in piglets with low and high SINS scores. Thirty-six genes were significant at *P* ≤ 0.05 in the offspring of different boars (upper part), 9 genes tended to be significant at *P* ≤ 0.1 (lower part)

					Expression in			
Gene Name	SSC	Position Start	Position End	Boar	SINSlow	SINShigh	Fold-increase	*P*
*ASAH2*	14	99,005,785	99,153,700	DU	103.7	460.1	4.4	0,0033
*ASAH2*	14	99,005,785	99,153,700	PI	877.6	430.7	-2.6	0,0056
*CCDC28A*	1	25,645,369	25,664,905	DU	270.4	86.1	-3.1	0,0001
*CCL20*	15	129,172,410	129,176,402	DU	93.2	33.7	-2.8	0,0338
*CFAP20*	6	19,953,229	19,970,852	DU	316.7	130.0	-2.4	0,0030
*CIRBP*	2	77,184,387	77,195,281	DU	236.8	578.8	2.4	0,0398
*FUS*	3	17,313,763	17,327,836	DU	347.7	999.1	2.9	0,0004
*GATM*	1	126,360,294	126,380,379	DU	1751.2	630.2	-2.8	0,0135
*GRAMD1C*	13	145,986,933	146,116,590	DU	661.8	271.6	-2.4	0,0352
*GTSF1*	5	19,637,081	19,664,018	DU	24.4	79.9	3.3	0,0443
*GYPA*	8	84,034,622	84,070,048	PI	565.3	139.7	-4.1	0,0095
*HAMP*	6	44,785,719	44,787,447	DU	80.0	350.4	4.4	0,0307
*HEMGN*	1	239,786,096	239,838,975	PI	492.8	161.8	-3.0	0,0343
*HNRNPDL*	8	135,801,796	135,810,605	DU	433.0	1064.1	2.5	0,0001
*IGSF5*	13	203,320,687	203,377,059	All	79.0	197.8	2.5	0,0068
*LIF*	14	47,219,743	47,241,310	DU	40.2	98.5	2.5	0,0480
*LOC100154071 (H2AC14)*	7	21,592,937	21,593,433	DU	823.0	319.2	-2.6	0,0142
*LOC100156277*	14	100,675,726	100,713,366	PI	97.1	33.6	-2.9	0,0043
*LOC100156618*	1	261,466,482	261,493,913	DU	405.2	1218.6	3.0	0,0180
*LOC100516628*	8	66,375,356	66,397,138	All	343.2	82.7	-4.1	0,0025
*LOC100516628*	8	66,375,356	66,397,138	DU	343.2	82.7	-4.1	0,0052
*LOC100517800(H2AB1)*	19	125,277,575	125,280,752	All	2203.9	911.6	-2.4	0,0078
*LOC100621538(H2AC25)*	2	51,688,375	51,704,824	DU	188.5	66.6	-2.8	0,0418
*LOC100623111*	2	8,619,513	8,625,035	All	96.9	315.5	3.3	0,0031
*LOC100736710*	5	20,648,836	20,652,451	DU	27.8	11.7	-2.4	0,0058
*LOC100737436*	4	44,237,395	44,238,625	DU	324.8	826.7	2.5	0,0008
*MGST2*	8	87,310,542	87,402,201	DU	628.3	123.7	-5.1	0,0065
*NAV2*	2	87,402,201	40,165,499	DU	144.0	419.7	2.9	0,0498
*P4HA1*	14	75,818,015	75,928,313	DU	1686.2	640.9	-2.6	0,0028
*RAPH1*	15	75,818,015	75,818,015	DU	608.0	1487.9	2.4	0,0500
*RDH16*	5	22,250,246	22,268,306	PI	1048.3	473.2	-2.6	0,0079
*S100A12*	4	96,225,557	96,227,164	DU	182.5	58.1	-3.1	0,0400
*SERPINA11*	7	115,664,489	115,664,489	DU	828.8	3803.0	4.6	0,0169
*SLC16A6*	12	11,856,083	11,877,066	DU	269.7	1024.7	3.8	0,0046
*SLC4A1*	12	18,956,068	18,977,011	PI	282.2	88.9	-3.2	0,0120
*TFRC*	13	133,974,686	133,999,633	DU	1554.1	647.3	-2.4	0,0009
*TMEM52B*	5	61,778,932	61,789,506	PI	955.5	284.5	-3.4	0,0263
*TMEM52B*	5	61,778,932	61,789,506	All	793.1	278.0	-2.9	0,0330
*VEGFA*	7	38,744,804	38,763,871	DU	78.4	191.1	2.4	0,0122
*CCL2*	12	38,763,871	40,800,204	DU	120.6	45.9	-2.6	0,0565
*CPNE8*	5	70,365,018	70,687,049	DU	438.4	146.0	-3.0	0,0604
*CRP*	4	70,687,049	90,801,786	PI	455.3	1687.7	3.7	0,0680
*GYPA*	8	84,034,622	84,070,048	DU	330.6	135.9	-2.5	0,0600
*GYPA*	8	84,034,622	84,070,048	All	444.0	100.7	-4.4	0,0944
*HAL*	5	87,519,982	87,549,990	DU	415.3	1091.6	2.6	0,0671
*JCHAIN*	8	67,438,483	67,449,550	DU	142.1	49.1	-2.9	0,0930
*LIPI*	13	179,123,343	179,185,818	DU	435.1	129.0	-3.4	0,0542
*LOC100525856(SAA1P)*	2	40,961,342	40,962,170	DU	682.5	1777.9	2.6	0,0810
*LY96*	4	61,916,715	61,953,803	DU	93.3	37.1	-2.5	0,0636
*SCD*	14	111,459,913	111,479,680	DU	346.1	1097.3	3.2	0,0742

P: significance; PI: Offspring from Pietrain boar; DU: Offspring from Duroc boar; SSC: Sus scrofa chromosome. Fold-changes are based on delogarithmised data

**Fig. 4:**
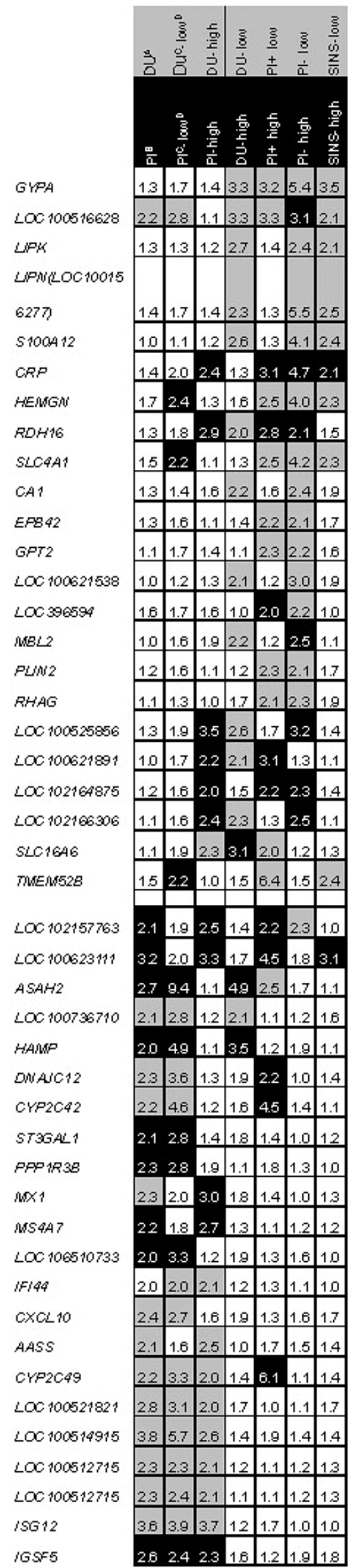
Fold changes of genes differentially expressed in liver tissue between different groups. Grey: groups of the grey head line (**A**) are up-regulated; black: groups of the black head line (**B**) are up-regulated. The left three columns represent more boar (“breed”) related, the right four columns represent more SINS-related genes. Genes are sorted with decreasing degree of SINS-relation and increasing degree of boar (“breed”) relation. **C**) Boars: DU = Duroc; PI + = relatively SINS stable Pietrain; PI- = realtively SINS susceptible Pietrain. **D**) low = low SINS scores (z-scores: -13.86 to -5.39), high = high SINS scores (z-scores: 4.18 to 12.83). Fold-changes are based on delogarithmised data

Figure 4 shows the detailed assignment of the differentially expressed genes to the compared groups, e.g. DU vs. PI (column 1), DU SINS-low vs. PI SINS-low (column 2) and DU and PI, each with pronounced SINS signs (SINS-high)(column 3). Expression differences are expected to show preferably boar differences that can be attributed to the breed, because the comparisons always take place within the same SINS level. However, no fundamental statement regarding the breed is to be made. Differentially expressed genes that fulfil this requirement are found especially in the lower half of the figure (*n* = 22). In contrast, the comparisons in columns 4 to 7 represent SINS effects, because in each case, progeny of low with progeny of high SINS levels are compared within a boar (DU: column 4, PI+; column 5; PI-: column 6) or across all boars (column 7). Only differentially expressed genes with multiple (at least twice) 2-fold changes or higher were included. From top to bottom, the involvement of SINS-related genes decreases in favor of primary boar related genes. Genes that are differentially expressed, especially under the influence of SINS, include *GYPA*, lipase k (*LIPK)*, lipase N (*LIPN)*, *S100A12*, *CRP*, *HEMGN*, retinol dehydrogenase 16 (*RDH16)*, *EPB42* and *TMEM52B*. In contrast, the genes immunoglobulin superfamily members 5 (*IGSF5)*, interferon stimulated gene 12 (*ISG12)*, some cytochrome genes, C-X-C motif chemokine 10 (*CXCL10)*, MX dynamin like GTPase 1 (*MX1)* and others reveal differences between boars that are not primarily associated with different SINS sensitivities. The numbers in the black boxes show the fold superiority of the subjects indicated in row 2, those in the grey boxes show the degree of up-regulation of the subjects indicated in row 1. *GYPA* and *CRP* are particularly prevalent among the SINS comparisons, *IGSF5* and *ISG12* among the more SINS-independent boar comparisons.

### Identification of biological processes and pathways

**Fig. 5:**
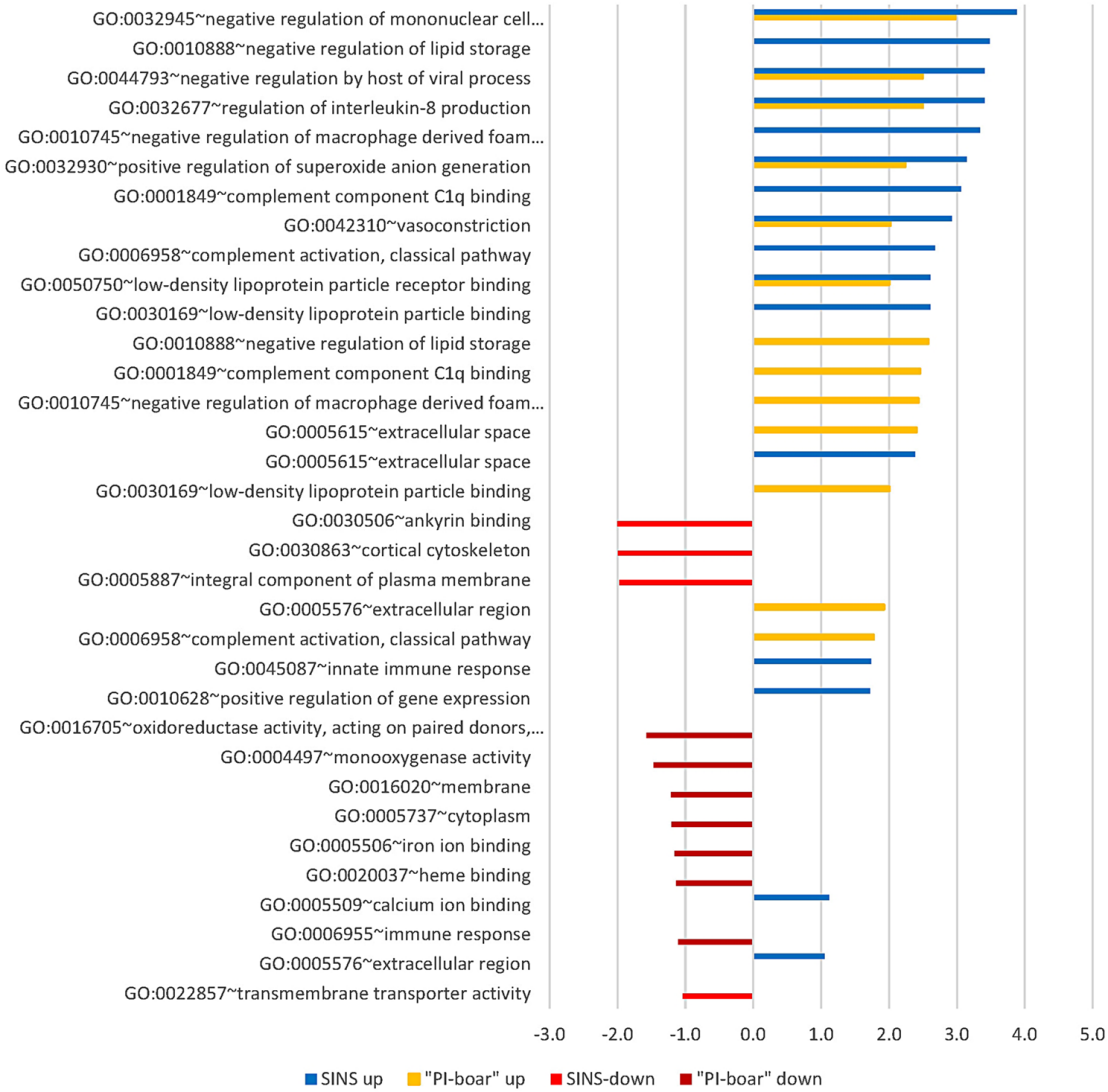
GO-terms and relative pathways up- (to the right) or down-regulated (to the left) with regard to SINS score and boar. SINS up: Up-regulated in pigelts with high SINS scores (z-scores from 4.18 to 12.83) compared to piglets with low SINS scores (z-scores from − 13.86 to -5.39). PI-boar up: Up-regulated in piglets from Pietrain boars as compared to piglets from Duroc boars

The genes of Fig. 4 were assigned to a whole series of interesting gene ontological pathways (Fig. 5). The pathways up-regulated under SINS in particular were largely consistent with the pathways up-regulated in PI boars. They are associated with the negative regulation of mononuclear cells and macrophages in particular, the negative regulation of viral processes by the host, and the negative regulation of IL8. Pathways for the activation of the complement system and vasoconstriction were also included. A pathway to innate immune response was up-regulated rather SINS-specifically. Down-regulated under SINS were pathways for ankyrin binding, concerning the cortical cytoskeleton and the plasma membrane. Some pathways were down-regulated in Pietrain without substantially affecting SINS. These pathways primarily involved haem and iron binding, cytoplasm and membrane. Some of the significant genes could not be assigned to any pathway.

### Verification of transcriptomic data

From the 44 genes of Table 2 with significant fold change in all or at least one group of offspring, seven genes were selected to be verified by qPCR. The expression of *GYPA*, *VEGFA*, *CCL2*, *CRP*, *IGSF5*, *S100A12* and *TMEM52B* was studied in liver tissue of suckling piglets. Effects of the boar (DU, PI+, PI-) and the SINS score (SINS-low vs. SINS-high) on the fold change compared to the general mean including all piglets was tested (Table 3). *VEGFA*, *TMEM52B*, and *GYPA* explained 27.8 to 35% of variance of expression. Expression was significantly down-regulated by SINS in *GYPA*, *S100A12* and *CCL*2 and up-regulated in *VEGFA* and *TMEM52B*. *IGSF5* was tendentially affected by breed and SINS and *CRP* was tendentially affected by SINS score.

**Table 3:** Effects of boar and SINS on average fold-expression of selected genes in qPCR

Gene		Total	DU	PI+	PI-	SINS-high	SINS-low	*P*_Boar_	*P*_SINS_	*R* ^2^_Boar_	*R* ^2^_SINS_
*GYPA*	Mean	2.06	1.89	1.98	2.31	0.62	3.50	n.s.	0.029	0.40%	27.80%
	SE	0.60	0.98	1.10	1.03	0.86	0.83				
*VEGFA*	Mean	1.26	1.32	1.12	1.33	1.70	0.81	n.s.	0.008	1.50%	35%
	SE	0.15	0.24	0.27	0.25	0.21	0.20				
*CCL2*	Mean	1.52	1.61	1.90	1.03	0.94	2.10	n.s.	0.029	5.30%	13.90%
	SE	0.33	0.54	0.61	0.57	0.485	0.46				
*CRP*	Mean	1.59	1.66	1.29	1.81	2.25	0.93	n.s.	0.060	1%	10%
	SE	0.49	0.81	0.91	0.85	0.71	0.69				
*IGSF5*	Mean	4.16	0.91	3.24	8.34	6.20	2.13	n.s.	n.s.	15.90%	6.60%
	SE	1.70	3.01	3.01	2.81	2.35	2.46				
*S100A12*	Mean	1.52	1.59	1.03	1.57	0.76	2.03	n.s.	0.030	0%	22.10%
	SE	0.36	1.43	0.60	1.84	0.35	1.68				
*TMEM52B*	Mean	2.97	2.28	3.08	3.56	5.04	1.79	n.s.	0.043	3.10%	34.97%
	SE	0.67	1.05	1.31	1.10	0.97	1.09				

SINS-high: Piglets with highest SINS-scores (z-scores between − 13.86 and − 5.39); SINS-low: piglets with lowest SINS-scores (z-scores between 4.18 and 12.83). P: significance; DU: offspring of the Duroc boar; PI-: offspring of the unfavourable Pietrain boar; PI+: offspring of the favourable Pietrain boar; SE: Standard error. R^2^: Coefficient of determination. Fold-changes are based on delogarithmised data

**Table 4:** Comparison of significant differentially expressed genes of the present study vs. Ringseis et al. [9]

	SINS Fold	MaxFold	DU vs.	DU low vs.	DU high vs.	DU low vs.	PI + low vs.	PI- low vs.	SINS-low vs.
Gene	Ringseis et al., 2021 [9] (^A^)	this study (abs)^B^	PI^C^	PI low	PI high	DU high	PI + high	PI- high	SINS-high^D^
*ADGRG6*	1.97	2.16	-1.39	1.16	-1.83	1.08	-2.16	-1.61	-1.57
*AKR1C4*	1.49	2.11	1.49	2.11	1.02	1.43	-1.78	-1.16	1.07
*CCL2*	-1.91	2.45	1.13	1.60	-1.81	2.45	-1.34	-1.08	1.27
*CDO1*	-1.77	2.57	-1.21	-1.15	-1.10	-1.49	-1.02	-2.57	-1.48
*CRP*	1.96	4.66	-1.42	1.97	-2.38	1.26	-3.13	-4.66	-2.06
*CYP1A2*	2.78	2.10	1.20	-1.27	1.89	-2.10	1.16	1.11	-1.24
*DGAT2*	1.34	2.19	-1.07	-1.56	1.41	-1.25	2.19	1.39	1.29
*ECHDC1*	1.30	2.90	1.15	1.54	-1.15	1.26	1.02	-2.90	-1.06
*EPB42*	2.25	2.21	-1.27	-1.62	-1.10	1.39	2.21	2.08	1.71
*ESM1*	2.70	2.11	1.36	1.27	1.20	1.58	1.07	2.11	1.59
*GYPA*	2.43	5.35	-1.29	-1.70	-1.39	3.30	3.22	5.35	3.51
*HEMGN*	2.03	3.99	-1.70	-2.39	-1.26	1.61	2.47	3.99	2.30
*INSIG1*	1.64	2.53	1.64	1.10	2.53	-1.68	1.14	1.67	-1.01
*JUN*	-1.58	3.03	-1.13	1.05	-1.09	-1.48	-3.03	1.12	-1.62
*LOC100156587*	1.35	2.07	1.00	-1.24	1.33	-1.68	-2.07	1.82	-1.24
*LOC100518644*	1.44	2.02	-1.07	1.32	-1.52	1.37	-1.09	-2.02	-1.13
*LOC100736962*	2.11	2.53	1.46	2.34	1.18	-1.05	-2.53	-1.66	-1.32
*LOC100737684*	1.43	2.26	-1.63	-2.26	-1.12	-1.84	1.36	-1.06	-1.22
*LOC733635*	2.30	2.07	1.06	-1.43	1.67	-1.53	1.25	2.07	1.10
*MUC13*	-2.02	2.05	-1.36	-1.78	1.00	-1.62	2.05	-1.90	-1.16
*PLN*	-1.52	2.40	-1.03	1.20	-1.25	1.14	1.11	-2.40	-1.12
*RHAG*	1.67	2.30	-1.06	-1.33	1.00	1.66	2.08	2.30	1.93
*SLC16A6*	-1.91	3.11	1.13	-1.91	2.30	-3.11	2.04	-1.22	-1.29
*SLC51B*	-2.28	2.00	-1.01	1.03	1.05	-1.27	1.00	-2.00	-1.25
*SPTA1*	1.46	2.64	-1.40	-1.93	-1.04	1.16	1.84	2.64	1.66
*UQCR10*	-3.02	2.31	-1.99	-2.31	-1.64	-1.32	1.45	-1.08	-1.16

^A^) Fold changes between piglets with low compared to piglets with high SINS scores (Ringseis et al.[9]); ^B^) Maximum of absolute Fold changes, present study; ^C^) Fold change between Duroc and Pietrain progeny, present study; DU: progeny of Duroc boar, this study; PI+: progeny of favourable Pietrain boar, this study; PI-: progeny of unfavourable Pietrain boar, this study; D) Fold changes between piglets with low SINS scores (z-scores between − 13.86 and − 5.39) and piglets with high SINS scores (z-scores between 4.18 and 12.83), this study. Fold-changes are based on delogarithmised data

## Discussion

SINS is a syndrome in pigs characterized by inflammation and necrosis of various parts of the body that can lead to pain, suffering and damage. Various studies showed signs at the base of the tail, tail tip, ears, teats, navel, coronary bands, claws and heels (for review see [6]). The syndrome starts with inflammatory loss of bristles, swelling, and redness. Later, rhagades, exudation, and possibly necrosis occur. The inflammation was shown histopathologically in newborn piglets, suckling piglets, weaners and fattening pigs [3–5]. Severe vasculitis, vascular thrombosis and lymphoplasmacellular inflammation [4] seem to be associated with a shortage of supply of the downstream tissues [1] in the sense of ischaemia with regard to the pigelts’ tails [12, 13].

The basal importance of inflammation was confirmed at various levels of clinical chemistry, metabolic, and transcriptomic findings [9, 11]. Signs of inflammation were found in the liver of affected animals. In three-day-old suckling piglets, mRNA levels of *FGF21*, *HP*, and *IL6* were elevated as a sign of the onset of an acute phase reaction. Increased *ICAM1*, *TNF*, and reduced *IL8* mRNA levels were indicative of stimulation of an inflammatory response. Overall, there was a consistent picture of increased numbers of monocytes and neutrophils, altered blood coagulation in weaners and thrombocytopenia in fatteners, as well as increased acute phase proteins, altered serum metabolites and increased serum liver enzymes [11].

The current study was based on progeny of one Duroc, one SINS-stable and one SINS-labile Pietrain boar mated to Topics-DL (DL: German Landrace) sows. In the comparative study by Ringseis et al. [9] Pietrain boars had been mated to Baden-Wurttemburg Genetics hybrid sows. Despite the different genetics of the tested piglets and the different experimental stalls and husbandry conditions, there was a concordance of 26 differentially expressed genes in both studies (Table 4). Among these genes were *CRP*, *GYPA*, *HEMGN*, *CCL2*, *EPB42* and others.

SINS was associated with elevated serum and liver mRNA levels of C-reactive protein (*CRP*) in recent studies [9, 11]. These results are confirmed by the present study under the conditions of the different husbandry and changed sow genetics, although the association was only significant at the *P* = 0.068 level. *CRP* was especially up-regulated in the offspring of Pietrain boars under SINS. However, in the Duroc progeny with SINS no significant role to *CRP* could be assigned. The up-regulation of *CRP* in SINS was confirmed by qPCR, again with a slightly lower P value of 0.06. The P values show a clear trend towards association between *CRP* and SINS, confirming previous studies and the physiological role of *CRP*. Therefore, it seems highly important to consider CRP in the context of SINS. Acute phase proteins are generally part of the systemic acute phase response and components of the innate immune system. The concentration of acute phase proteins in the plasma is altered in animals subjected to challenges such as infection, inflammation, trauma or stress [14]. Their synthesis takes place mainly in the liver under the stimulus of the pro-inflammatory cytokines interleukin 1ß (*IL1β)*, *IL6* and *TNF* [15], which were found elevated in SINS in former studies [9, 11]. Among the acute phase proteins, *CRP* was more informative than *HP* [11]. In some studies, *CRP* was the only clearly increased acute phase protein in serum in pigs [16], sometimes with delay [17]. As an acute-phase serum protein and a mediator of innate immunity, *CRP* binds to microbial polysaccharides and to ligands exposed on damaged cells. Binding of *CRP* to these substrates activates the classical complement pathway leading to their uptake by phagocytic cells [18]. *CRP* is involved in several host defence related functions based on its ability to recognize foreign pathogens and damaged cells of the host and to initiate their elimination by interacting with humoral and cellular effector systems in the blood. Consequently, the level of this protein in plasma increases greatly during acute phase response to tissue injury, infection, or other inflammatory stimuli. It promotes agglutination, bacterial capsular swelling, phagocytosis and complement fixation through its calcium-dependent binding to phosphorylcholine [19]. Elevated levels of serum *CRP* and *CRP* expression agree well with the clinical and histopathological findings of vasculitis, intima proliferation and thrombosis in SINS [3, 4] and support the assumption of SINS as a vascular disease. Elevated *CRP* levels were also described in artherosclerosis [20]. Potentially significant polymorphisms in the 3‘ UTR region of porcine *CRP* had been associated with variation in serum CRP-levels [21, 22]. However, the exact cause of the increased *CRP* values in the present study remains open.

One further interesting differentially expressed candidate gene related to SINS is the phagocytotic proinflammatory protein *S100A12*. This protein was described as a damage-associated molecular pattern [23] because it plays an important role in the context of inflammation, innate immunity and antimicrobial defense [24]. This places *S100A12* among the critical components of the innate immune system. However, it is involved in a complex regulatory system that has not yet been completely deciffered. In a number of gastrointestinal inflammatory diseases, such as necrotizing enteritis, inflammatory bowel disease, Crohn’s disease, irritable bowel syndrome, *S100A12* levels are significantly elevated in affected tissues [25]. Similar results were described with relation to a wide range of inflammatory and infectious diseases of other organ systems [24]. Associations between *S100A12* levels and different disease patterns have also been shown for different diseases in swine [26]. *S100A12* emerged as one of the most conspicuous differentially expressed genes in the present study. There were significant associations between *S100A12* and SINS in the progeny of the Duroc boar and the Pietrain boar classified as unfavourable. However, *S100A12* was down-regulated in the piglets with pronounced SINS findings. As a possible explanation, Chen et al. [26] state that the overexpression of pro-inflammatory cytokines as a result of *S100A12* action can lead to drastic tissue damage (or even septicaemic shock) and that the expression of *S100A12* is tightly regulated. The authors present findings of negatively regulating elements within the promoter region of *S100A12* [26]. Supplemental to this, Wu et al. [27] show that *S100A12* may even have an inhibitory function in inflammation. They could show that after blockade of *S100A12* the expression of *TNF* and *IL10* even increased and postulated that *S100A12* competes with lipopolysaccharides (LPS) for the binding site at toll-like receptor 4 (TLR4), but activates it more weakly than LPS itself. Thus, LPS signaling at TLR4 might be more inhibited in the SINS-stable pigs of the present study than in the more SINS-sensitive pigs because of higher *S100A12* expression in the former, explaining higher levels of inflammation in the latter. Another explanation for a higher expression of *S100A12* in animals with lower inflammation rates in the current study could be the momentary nature of the study. The starting point for the inflammation in the individuals could not be determined. Chen et al. [26] presented a maximum curve in the progression of *S100A12*, which was followed by a delay of six to twelve hours in different breeds of pigs, so that one breed initially had higher, and later lower, levels than the other. Exact ideas on the course of *S100A12* levels after activation or in the chronic case are not yet available. Taken together, to clear the context of the present findings, several questions regarding gene regulation in *S100A12* in general and specifically in pigs remain to be clarified.

The chemokine *CCL2* was found down-regulated in relation with high SINS scores. It is involved in immunoregulatory and inflammatory processes. *CCL2* displays chemotactic activity for monocytes and basophils [28]. An earlier name for *CCL2* was monocyte chemoattractant protein-1 gene (*MCP1*). It has been implicated in the pathogenesis of diseases characterized by monocytic infiltrates, like psoriasis, rheumatoid arthritis and atherosclerosis [29]. *CCL2* signals through binding and activation of the C-C motif chemokine receptor 2 (CCR2) and induces a strong chemotactic response and mobilization of intracellular calcium ions [30]. *CCL2* seems to be involved in the recruitment of monocytes into the arterial wall during the disease process of human atherosclerosis [31]. As vasculitis and elevated monocyte numbers are typical findings of SINS [4, 9, 11], *CCL2* arises as a strong candidate gene in SINS pathogenesis. Different gene variants (cis or trans) might lead to higher monocyte recruitment rates followed by higher degrees of SINS. It was shown that porcine *CCL2* mechanisms works in the same way than in mouse and human and that porcine monocyte subsets differ in the expression of *CCR2* and in their responsiveness to *CCL2* [32].

Another chemokine which is differentially expressed in SINS is *CXCL10*, a pro-inflammatory cytokine that is involved in a wide variety of processes such as chemotaxis, differentiation, and activation of peripheral immune cells, regulation of cell growth, apoptosis and modulation of angiostatic effects [33]. Binding of this protein to C-X-C motif chemokine receptor 3 (CXCR3) results in pleiotropic effects, including stimulation of monocytes, natural killer and T-cell migration, and modulation of adhesion molecule expression. Binding of *CXCL10* to the CXCR3 receptor activates T-helper 1 (TH1) lymphocytes and is crucial in directing activated T cells to sites of inflammation. As such, they play an important role in several chronic inflammatory diseases including ulcerative colitis and artherosclerosis [34]. *CXCL10* expression was different in longissimus dorsi muscle of different pig breeds [35]. Again, the exact potential role of *CXCL10* in the pathogenesis of SINS remains to be clarified.

*VEGFA* was found up-regulated by SINS in the present study. It induces proliferation and migration of vascular endothelial cells, and is essential for both physiological and pathological angiogenesis. It induces permeabilization of blood vessels [36]. Disruption of this gene in mice and humans results in pathogenic stati. Allelic variants of this gene have been associated with microvascular complications of diabetes 1 (MVCD1) and atherosclerosis. Alternatively, spliced transcript variants encoding different isoforms have been described. There is also evidence for alternative translation initiation from upstream non-AUG (CUG) codons resulting in additional isoforms. A recent study showed that a C-terminally extended isoform is produced by use of an alternative in-frame translation termination codon via a stop codon readthrough mechanism, and that this isoform is antiangiogenic. Increasing levels of *VEGF* during infection can promote inflammation by facilitating recruitment of inflammatory cells together with angiotensin-converting enzyme 2 (ACE2). *VEGFA* is also expressed as a result of hypoxia [37], which is thought to be a central element in the pathogenesis of SINS in piglets [1, 4, 13]. Increased angiogenesis has been shown by histopathology (Wenisch, pers. communication). *VEGF* plays a pivotal role in angiogenesis in ovaries, particularly during follicular development and ovulation. Interleukin-6 (IL6) is one of the major pro-inflammatory factors that are involved in the angiogenesis process physiologically and pathologically and the high level of prostaglandin E2 (PGE2) causes an inflammatory-like microenvironment before ovulation. This response seems to be involved in fertility in swine [38] and might be a reason for the selection towards certain *VEGFA* variants, eventually with consequences for their reactivity in angiogenesis. Further studies are needed to understand the exact context of this possible relation.

GYPA is a sialoglycoprotein and the major intrinsic membrane protein of the human erythrocyte membrane. The N-terminal glycosylated segment, which lies outside the erythrocyte membrane, has MN-blood group receptors. It appears to be important for the function of *SLC4A1* and it is a receptor for influenza virus and other pathogens [39]. *GYPA* was increased in expression independent of breed as well as in the Pietrain breed by 4-fold, and in DU by 2.5-fold in the SINS-stable piglets compared with the expression of the SINS-sensitive piglets. The pronounced differential expression was striking. Nevertheless, it remains unclear what role these differences might play with respect to sensitivity to SINS. The relation between *GYPA* and *SLC4A1* becomes also visible in the present study. We found higher levels of expression in Pietrain offspring than in offspring of the DU boar, but a down-regulation in SINS. *SLC4A1* [40] is also involved in the membrane of the erythrocyte and can be responsible for genetic erythrocyte disorders. A third blood group protein in swine is *EPB42* [41] and like *GYPA* it was found down-regulated in piglets with high SINS scores. The surprisingly clear involvement of erythrocyte antigens and genes involved in their metabolism, together with the appearance of the typical SINS-signs in the area of the acras, raises the suspicion of an increased susceptibility of the erythrocytes in the affected animals to cold agglutinins. Warm-, cold-, or mixed-reactive antibody types that are directed against antigens on the red blood cell (RBC) surface can lead to hemolytic anemia in humans [42]. The autoantibodies may be idiopathic or related to an underlying condition such as infection, malignancy, or immune disease. In pigs, cold agglutinins have been described in association with inflammation of the ears in *Mycoplasma suis* infections [43] (which were excluded for the present study). Signs of haemolytic anaemia have not yet been observed in connection with SINS. However, it is possible that inflammation- or infection-associated antibodies may contribute to the described vasculitis and thrombosis via interaction with erythrocyte epitopes without causing measureable haemolytic anaemia. However, the above-mentioned context should be a reason for further research.

Finally, the *LIPK* and *LIPN* [44] genes should be mentioned, whose products are involved as lipases in the lipid metabolism of keratinocytes and as such are responsible for stable keratinisation of the epidermis. Both were down-regulated in piglets with a high SINS score. This could speak for an additional weakening influence on the stability of the epidermis, in line with the assumption that under SINS a higher susceptibility of the exposed tissues to environmental conditions, such as unfavourable floor conditions, arises [4].

Limitations of the study arise from the fact that the generally useful consideration of the significance level after multiple testing was not included in the present study. This approach is not uncommon for hybridisation-based transcriptome analyses (e.g [9, 47–49]), which are basically only of a screening nature. If the number of replicates is very small due to the experimental design, it will hardly be possible to detect effects after significance correction due to multiple testing. Therefore, qPCR validations were performed to corroborate the screening results and associate them with clinical features. In this way, indications of candidate genes were obtained that are in a homologous-physiological association with the SINS clinic.

## Conclusion

The present study highlights the inflammatory nature of SINS. It confirms the importance of genes of inflammation and defence, such as *CRP* in the SINS process and adds a whole series of further candidate genes such as *S100A12*, *GYPA*, *LIPK*. In addition, there are indications of a possible disturbance of the keratin metabolism in sensitive and affected animals. The detection of these gene products could be used for the diagnosis of SINS in the future. In addition, the genes provide valuable starting points for a comprehensive elucidation of the pathogenesis and for combating the syndrome, thus improving animal welfare.

## Methods

### Study design

The animal experiment was carried out in the conventional pig breeding stables of the Oberer Hardthof teaching and research station at Justus-Liebig University Giessen under the approval of the responsible animal welfare office of the Justus-Liebig-University in Giessen, Germany (file number 708_M). All methods were carried out in accordance with relevant guidelines and regulations. The approval involved a protocol including the research question, key design features, and analysis plan which was prepared before the study.

Sows and suckling piglets were kept in 4.8 m^2^ farrowing pens with a plastic slatted floor. The sows were fixed in a farrowing crate with a flat surface. The sow´s floor was a slatted cast iron floor. The feed was offered via a spotmix feeder in the trough. Nipple drinkers and mother-child basin drinkers ensured the water supply for the animals.

The feed for nursing sows consisted of wheat (36.5%), barley (31.0%), HP soybean extraction meal (15%), beer yeast (2.0%), fiber mix (7.5%, Ingredients: wheat bran, beet molasses pulp, sunflower seed extraction meal, oat hulling bran, lignocellulose, lucerne flour, malt culms, beet molasses), a mineral feed mixture (3.5%; 5% lysine, 2,3% methionine and cysteine, 2.5% threonine, 0,2% thryptophane, 19% calcium, 4.5% phosphorus, 5.3% sodium), an energy mix (0.4%) a herbal mix (0.5%, inter alia 15% fenugreek seed, 5% milk thistle seed, apple pomace, oyster shell lime, carrots, malt culms, yeast) and an acid mix (0.4%). The energy content of the lactation diet was calculated to 12.45 MJ ME/kg and the crude protein was 16.5%.

The sow herd (Topigs x German landrace) was artificially inseminated with three extreme boars based on the study by Kühling et al. [5]. Boars were used pairwise as mixed semen to have piglets from two different boars present in each litter at the same time. The design was applied to (i) limit the number of litters and experimental animals, (ii) to minimise environmental effects, (iii) to increase genetic variability within the piglets, (iv) to increase the sow-boar combinations and (v) to nevertheless achieve a manageable number of piglets.

The three boars were a Duroc boar (DU) and two extreme Pietrain boars (PI + and PI-) who’s progeny had SINS scores of 10.4 (Confidence interval: 9.3–11.6), 12.7 (CI: 12.1–13.2), and 13.8 (CI: 13.2–14.4), respectively. These boars were selected to achieve segregation of favourable and unfavourable gene variants in the progeny. All sows were used only once. They were inseminated with mixed semen from two boars, so that piglets of the Pietrain boar classified as unfavourable (PI-) occurred in one litter together with piglets of the Duroc boar (DU) (in 13 litters) or together with piglets of the Pietrain boar classified as favourable with respect to SINS (PI+) (in 14 litters). This was done to minimise the environmental effects on the SINS-outcome of the piglets. Thus, 27 sows produced 27 litters. 236 piglets were available, as long as they did not have any anatomical aberrations. Their SINS phenotypes were recorded on their third day of life. The piglets’ father was detected by paternity testing after phenotyping [45]. The results of paternity testing revealed 14 mixed litters (from 14 sows) with 77 piglets from the favourable Pietrain boar and 39 piglets from the unfavourable Pietrain boar, as well as 13 mixed litters from 13 sows with 48 piglets from the Duroc boar and 70 piglets from the unfavourable Pietrain boar. On average, 8.4 healthy piglets per litter with at least one piglet per inseminated boar were sampled.

### Sample collection

The piglets were anaesthetized intramuscularly with a mixture of 40 mg/kg ketamine (Ursotamin^®^ 10%, Serumwerk Bernburg AG, Bernburg, Germany) and 4 mg/mL azaperone (Stresnil^®^, Elanco, Indianapolis, IN, USA). The animals were euthanized with an intracardiac injection of 45 mg/kg pentobarbital sodium (Release^®^ 500 mg/mL, WDT Eg, Garbsen, Germany). Death was determined by auscultation of the heart with a stethoscope (double-headed stethoscope, Henry Schein GmbH, Melville, NY, USA), the absence of spontaneous respiration, and by testing the eyelid closure, cornea, nasal septum and interclaw reflexes.

The animals were placed on a stainless steel table (work table type KST-041, R&S Edelstahl und Technik GmbH, Hamm, Germany) with an absorbent pad (sick pad, Henry Schein GmbH, Melville, NY, USA). After death, the abdominal cavity of the animals was opened along the linea alba with a scalpel blade (Aesculap^®^ BB522, B. Braun AG, Melsungen, Germany). Approximately 1 g of material was removed from the liver at the lobus sinister medialis. Aliquots of the liver were excised, washed in sterile ice-cold NaCl solution (0.9%) and collected in microcentrifuge tubes (Sarstedt, Nümbrecht, Germany). The liver samples were immediately snap-frozen in liquid nitrogen and stored at -80 °C, until pending RNA extraction.

**Table 5:**
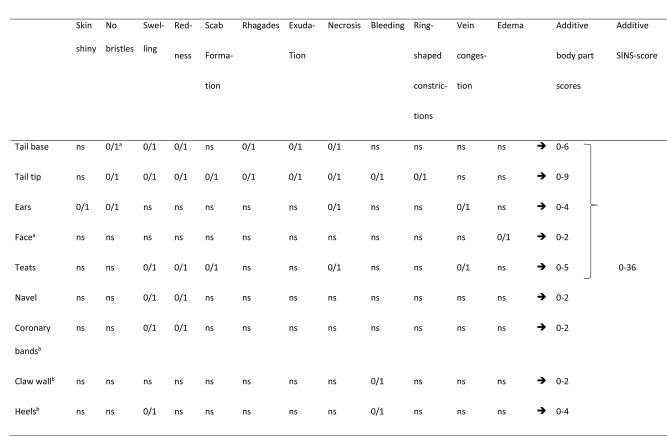
Overview of the collected individual phenotypic characteristics and scores

### Paternity testing

Genetic matches between offspring and boars were used in paternity testing. DNA was extracted from the piglets’ docked tails by using the smart DNA prep (m) kit (Analytik Jena, Jena, Germany) [45]. Samples were genotyped with 14 microsatellites in two multiplex PCRs and microsatellite alleles were determined by capillary gel-electrophoresis (ABI PRISM^®^ 310 Genetic Analyzer, Applied Biosystems, Thermo Fisher Scientific, Waltham, MA, USA).

### Clinical scoring

Inflammation and necrosis were clinically assessed as the major outcome measures (described by Reiner et al. [2]). The third day of life was chosen to ensure comparability with other studies and to minimize environmental effects. Clinical signs were clearly visible during this period in all previous studies, and the piglets were not yet as much exposed to environmental effects like weaners and fatteners. Clinical signs were recorded using a digital camera to minimize the animal load (Canon EOS DC 8.1 V, Canon, Krefeld, Germany) according to a standardized scheme for later detailed evaluation of the images (Windows Media Player, Version 12, Microsoft GmbH, Germany).

Clinical alterations in the tail base and tail tip, the ears, the teats and navel, coronary bands, wall horn, heels and sole of the feet as well as the face were assessed individually. Clinical characteristics were considered and scored 0, if the sign was absent or 1 if the sign was visible (Table 5). The tail base and tail tip were independently scored for loss of bristles, swelling, redness, scab formation (tail tip only), rhagades, exudation, necrosis, bleeding (tail tip only), and ring-shaped constrictions (tail tip only). Ears were scored for a shiny skin, the loss of bristles, necrosis and congested ear veins. The face was scored for the absence or presence of edema at the eye lids and nose back. Teats were scored for swelling, reddening, scab formation, necrosis and congested blood vessels. The navel was scored for signs of inflammation in the form of redness or swelling. Claws were scored qualitatively for any signs of inflammation at the coronary bands (swelling, redness or exudation), wall bleeding, as well as swelling and bleeding of the heels.

All binary signs were summed up to give an additive body part score (Table 5). Scores could reach 2 to 9 points. All scores were summed up for the SINS score in an unweighted manner. This resulted in possible SINS scores between 0 and 36 for each piglet. All scores were assigned by two experienced persons together. An overview on the evaluated phenotypes is given in Table 5. It was not possible to assign the piglets to their fathers at the time of clinical scoring because mixed semen was used and no paternity test was available at that time.

### Transcriptomics

#### Selection of piglets for transcriptomics

After paternity of the piglets had been determined and the SINS values calculated, the four offspring with the lowest (SINS-low) and four offspring with the highest (SINS-high) SINS values were selected from the DU, PI + and PI boar offspring for the comparative gene expression study. Together, liver samples from 24 piglets were used. The piglets were the experimental unit. They were three days old and 59% were female, 41% were male. The piglets weighed 1.35 ± 0.4 kg at birth.

#### RNA purification

Total RNA was extracted from 20 mg of porcine liver tissue according to the “Purification of total RNA from animal and human tissue” protocol of the RNeasy Micro Kit (QIAGEN, Hilden, Germany).

In brief, the tissue was stabilized in RNAlater-ICE reagent (Invitrogen, Thermo Fisher Scientific, Waltham, MA, USA) and shipped on dry ice. After removing the RNAlater-ICE, the tissue was disrupted and homogenized in 350 µl RLT buffer containing 1% beta-mercaptoethanol with Precellys CK14 ceramic beads (1 cycle of 20 s at 5000 rpm for the liver samples and two cycles of 20 s at 6500 rpm for the tail-base samples) using a Precellys 24 Homogenisator (Bertin Corp., Rockville, MD, USA). Next the sample was centrifuged for three min at full speed and 350 µl of the cleared supernatant was transferred to a new tube. One volume of 70% ethanol was added and the sample was applied to a RNeasy MinElute spin column followed by an on-column DNase digestion and several wash steps. Finally, total RNA was eluted in 14 µl of nuclease free water. Purity and integrity of the RNA was assessed on the Agilent 2100 Bioanalyzer with the RNA 6000 Nano LabChip reagent set (Agilent, Palo Alto, CA, USA). The average RNA concentration and RNA integrity number (RIN) of all total RNA samples (*n* = 18, means ± SD) was 454 ± 34 ng/µL and 8.0 ± 0.6, respectively.

#### GeneChip microarray assay

Sample preparation for microarray hybridization was carried out as described in the GeneChip Whole Transcript (WT) PLUS Reagent Kit User Guide (Applied Biosystems, Thermo Fisher Scientific, Waltham, MA, USA).

In brief, 300 ng of total RNA was used to generate double-stranded cDNA. 12 µg of subsequently synthesized cRNA were purified and reverse transcribed into single-stranded (ss) cDNA, whereat unnatural dUTP residues were incorporated. Purified ss cDNA was fragmented using a combination of uracil DNA glycosylase (UDG) and apurinic/apyrimidinic endonuclease 1 (APE 1) followed by a terminal labeling with biotin. 3,8 µg of fragmented and labeled ss cDNA were hybridized to Applied Biosystems GeneChip Porcine Gene 1.0 ST arrays which covers 19,212 genes represented by 394,580 probes, for 16 h at 45° C and 60 rpm in an Applied Biosystems GeneChip hybridization oven 640. Hybridized arrays were washed and stained in an Applied Biosystems GeneChip Fluidics Station FS450, and the fluorescent signals were measured with an Applied Biosystems GeneChip Scanner 3000 7G System. Fluidics and scan functions were controlled by the Applied Biosystems GeneChip Command Console v5.0 software.

Sample processing and Affymetrix microarray hybridization were carried out at the Genomics Core Unit: Center of Excellence for Fluorescent Bioanalytics (KFB, University of Regensburg, Germany).

#### Microarray data analysis

Summarized probe set signals in log2 scale were calculated by using the RMA algorithm [46] with the Applied Biosystems GeneChip Expression Console v1.4 Software and exported into Microsoft Excel. The Microarray study was MIAME compliant.

Following scanning of the processed GeneChips, cell intensity files, which provided a single intensity value for each probe cell, were generated from the image data using the Command Console software (Affymetrix). Correction of background and normalization of probe cell intensity data was carried out using the Robust Multichip Analysis (RMA) algorithm with the Expression Console software (Affymetrix). RMA algorithm is a log scale multi-chip analysis approach fitting a robust linear model at the probe level to minimize the effect of probe-specific affinity differences. Expression levels of transcripts are measured using log transformed perfect match values, after carrying out a global background adjustment and across microarray normalization. Annotation of microarrays was performed with the NetAffx annotation file “Porcine Annotations, CSV format, Release 36 (6.4 MB, 4/13/16)”. The reference genome used was Sus scrofa 11.1.

After quality control three to five offspring with particularly low (SINS-low) and with particularly high (SINS-high) SINS scores, each from the DU, the PI + and the PI- boar offspring could be further analysed. The exact numbers were: 5 DU-low, 3 DU-high, 3 PI+-low, 3 PI+-high, 3 PI—low, 4 PI—high. Together, liver samples from 21 piglets were used in the end.

Transcriptomic data as such generally suggest moderate differences rather than targeted single gene setups. They were therefore used in the present study in the sense of a screening for possibly involved genes. In order to discover genes differentially regulated in connection with SINS, fold changes <-2 and > 2 were used with *P* ≤ 0.05 in the unpaired Student’s t-test (two-tailed distribution, two-sample equal variance). Some further genes within *P* ≤ 0.1 were considered as tending to be significant. Identical or similar filter criteria were also applied in several recent studies [9, 47–49], in which treatment-induced changes of the transcriptome were only moderate and the application of more stringent filter criteria (e.g., false discovery rate and/or FC > 2.0 or <-2.0) failed to filter a substantial number of genes being sufficient to perform gene set enrichment analysis (GSEA). That is why qPCR validations were carried out to corroborate the screening results and associate them with the clinical features. The genes identified in this way can be analysed specifically in further targeted studies without the background of an entire transcriptome. Differentially expressed transcripts were processed using the freely available DAVID 6.8 bioinformatic resource [50] (https://david.ncifcrf.gov/) to identify enriched Gene Ontology (GO) biological process terms Biological process terms were considered as enriched if *p* < 0.05. This was carried out separately for the up- and down-regulated transcripts with regard to SINS and for up- or down-regulation in Duroc vs. Pietrain offspring, respectively, for easier interpretation of data. According to this approach, biological processes identified as enriched within up-regulated and down-regulated genes are assumed to be activated and inhibited, respectively.

#### Candidate gene prediction

Information on transcriptomic candidate genes was obtained with GeneCards [51, 52] (https://www.genecards.org/) a human gene data base. This program was used to detect genes involved in inflammation, vasculitis and necrosis. Genes were displayed in order of the Gene Card Score, where best fitting genes obtain the highest order.

Validation of microarray data using qPCR analysis.

For validation of transcriptomics by quantitative PCR (qPCR), 21 mRNA preparations were available from progeny of Duroc, Pietrain boar classified as favourable, and Pietrain boar classified as unfavourable, with the lowest and highest SINS scores, respectively.

The cDNA was synthesised with the High Capacity cDNA Reverse Transcriptase Kit (Applied Biosystems, Thermo Fisher Scientific, Waltham, MA, USA) using a Biometra T personal 48 Thermocycler (Biometra, Analytik Jena, Jena, Germany).

For seven target genes (*GYPA*, *VEGFA*, *CCL2*, *CRP*, *IGSF5*, *TMEM52B*, *S100A12*) and one housekeeping gene (*GAPDH*), gene-specific primer pairs were generated using Primer3 [53] and Basic Local Alignment Search Tool (BLAST) (Table 6). To avoid contamination with genomic DNA (gDNA), the primers span an exon-exon junction when possible. During primer validation, the primer concentration was optimally adjusted and the existence of possible primer dimers was excluded by melt curve analysis. The assay efficiency was calculated by running a 5-point standard curve, in a duplicate approach, using 10-fold dilutions.

The qPCR was performed on a QuantStudio 3 (Applied Biosystems, ThermoFisher Scientific, Waltham, MA, USA) using the PowerUp SYBR Green Master Mix (Applied Biosystems, Thermo Fisher Scientific, Waltham, MA, USA) Conditions: see Table 7.

The CT values were normalised analogously to the method of Pfaffl [54] using the housekeeping gene *GAPDH* and were corrected for the real-time PCR efficiency of the respective target genes. Since no explicit control was available in the present study, the overall mean of the CT values of all animals was used as the control value for the respective gene. Subsequently, the deviations of the CT values of the individual animals from the overall mean were exponentiated with the amplification factor and the effects of breed and SINS status on the result were analysed.

### Statistical analysis

Standard statistical analysis was done with IBM-SPSS, version 27 (IBM, Munich, Germany). Frequencies of binary data were calculated as means. Clinical scores for body parts and SINS are known to be normally distributed [2, 6]. They were z-transformed before analysis. Effects of Boar and SINS, including coefficients of determination for the metric distributed scores were calculated with two-factorial analysis of variance. Associations between gene expression data and SINS (low vs. high) respectively boar (DU vs. PI + vs. PI-) effects were calculated with Student’s T-test. SINS-low and SINS-high were defined as z-transformed values from − 13.86 to -5.39 and from 4.18 to 12.83, respectively. ‘The data for the 21 piglets were available as log2 values. They were delogarithmised and the fold changes between the groups (SINS, boar) shown in the tables and figures are based on these delogarithmised data.’To estimate the effects of SINS and boar on the qPCR results, a two-factorial analysis of variance was applied to the Fold-changes (e.g. SINS-low vs. SINS-high, DU-boar vs PI-boars etc.).

**Table 6:** Primers and PCR conditions for qPCR analysis

Genes	Forward-Primer	Reverse-Primer	Length (bp)	Annealing temperature (°C)	GC-content forward/ reverse (%)
*GYPA*	TTTTTGCAGTGATGGCTGGC	GGCACAGGCAAAGGTTTCTTT	88	60	50/47
*VEGFA*	CTTGCCTTGCTGCTCTACCT	TCCATGAACTTCACCACTTCGT	98	60	55/45
*CCL2*	TGCCCAGCCAGATGCAATTA	CCGCTGCATCGAGATCTTCT	76	60	50/55
*CRP*	CTGTCTATGCTGGTGGGACC	GCTTGACATACACCTCGCCA	87	60	60/55
*IGSF5*	CACAACTTCTCTGCCTCCGA	GCGGTGATCCAGTCCCTG	88	60	55/66
*TMEM52B*	TTCATCCAGCTTCCTTGGGC	CAGGCCACACAGCAGAAGTA	135	60	55/55
*S100A12*	GCCCAACACCCTCAAGAACA	CAGCACATCAGTCACCAGGA	124	60	55/55
*GAPDH*	CACCATCTTCCAGGAGCGAG	GCCTTCTCCATGGTCGTGAA	102	60	60/55

**Table 7:** Thermal cycling conditions: fast cycling (for Applied Biosystems QuantStudio instruments and primer tm ≥ 60 °C)

Step	Temperature (°C)	Time (in sec)	Cycles
*UDG activation*	50	120	Hold
*Polymerase activation*	95	120	Hold
*Denaturation*	95	1	40
*Annealing/extension*	60	30	
*Melt curve stage*			Ramp rate
*Step 1*	95	15	1.6 °C/sec
*Step 2*	60	60	1.6 °C/sec
*Step 3*	95	15	0.15 °C/sec

## Electronic supplementary material

Below is the link to the electronic supplementary material.

Supplementary Material 1

## Data Availability

The data discussed in this publication have been deposited in NCBI’s Gene Expression Omnibus [55, 56] and are accessible through GEO Series accession number GSE243954 (https://www.ncbi.nlm.nih.gov/geo/query/acc.cgi?acc=GSE243954).

## Abbreviations

ACE2: Angiotensin-Converting Enzyme 2
ALAS1: 5’-Aminolevulinate Synthase 1
ASAH2: N-Acylsphingosine Amidohydrolase 2
CCL2: C-C Motif Chemokine Ligand 2
CCR2: C-C Motif Chemokine Receptor 2
CRP: C-reactive protein
CXCL10: C-X-C Motif Chemokine 10
CXCR3: C-X-C Motif Chemokine Receptor 3
CYP2C42: Cytochrom p450 c42
CYP2C49: Cytochrom p450 c49
DL: German Landrace
DU: Duroc
EPB42: Erythrocyte Membrane Protein Band 4.2
FMO5: Dimethylaniline Monooxygenase 5
GYPA: Glycophorin A
HEMGN: Hemogen
HP: Haptoglobin
ICAM1: Intercellular Adhesion Molecule 1
IGSF5: Immunoglobulin Superfamily Members 5
IL10: Interleukin 10
IL1ß: Interleukin 1ß
IL6: Interleukin 6
IL8: Interleukin 8
ISG12: Interferon Stimulated Gene 12
JUN: Jun Proto-Oncogene
LIPK: Lipase K
LIPN: Lipase N
LPS: Lipopolysaccharides
MCP1: Monocyte Chemoattractant Protein-1
MVCD1: Microvascular Complications of Diabetes 1
MX1: MX Dynamin like GTPase 1
PGE2: Prostaglandin E2
PI: Pietrain
PI: SINS-Susceptible Pietrain
PI+: SINS-Resistant Pietrain
RBC: Red Blood Cell
RDH16: Retinol Dehydrogenase 16
S100A12: S100 Calcium Binding Protein A12
SINS: Swine Inflammation and Necrosis Syndrome
SLA: Swine Lymphocyte Antigen
SLC16A6: Solute Carrier Family 16 Member 6
SLC38A1: Solute Carrier Family 38 Member 1
SLC4A1: Solute Carrier Family 4 Member 1
SOD1: Sodiumoxid Dismutase 1
TH1: T-helper 1
TLR4: Toll-Like Receptor 4
TMEM52B: Transmembrane Protein 52B
TNF: Tumor Necrosis Factor
VEGFA: Vascular Endothelial Growth Factor A
